# MiR-30 Family Potentially Targeting PI3K-SIAH2 Predicted Interaction Network Represents a Novel Putative Theranostic Panel in Non-small Cell Lung Cancer

**DOI:** 10.3389/fgene.2017.00008

**Published:** 2017-02-02

**Authors:** Lawrence W. C. Chan, Fengfeng Wang, Fei Meng, Lili Wang, Sze Chuen Cesar Wong, Joseph S. K. Au, Sijun Yang, William C. S. Cho

**Affiliations:** ^1^Department of Health Technology and Informatics, Hong Kong Polytechnic UniversityHong Kong, Hong Kong; ^2^Hong Kong Adventist HospitalHong Kong, Hong Kong; ^3^ABSL-3 Laboratory at the Center for Animal Experiment and State Key Laboratory of Virology, School of Medicine, Institute of Animal Model for Human Diseases, Wuhan UniversityWuhan, China; ^4^Department of Clinical Oncology, Queen Elizabeth HospitalHong Kong, Hong Kong

**Keywords:** PI3K, SIAH2, non-small cell lung cancer, theranostics, microRNA

## Abstract

Non-small cell lung cancer (NSCLC) comprises about 84% of all lung cancers. Many treatment options are available but the survival rate is still very low due to drug resistance. It has been found that phosphoinositide-3-kinase (PI3K) affects sensitivity to tyrosine kinase inhibitors (TKIs), including gefitinib and erlotinib. Expression level of seven in absentia homolog 2 (SIAH2), an E3 ubiquitin-protein ligase, is upregulated in NSCLC and correlated with tumor grade. However, the relationship between PI3K and SIAH2 remains unclear and therefore it is not known whether they can act as treatment co-targets and theranostic dual markers for overcoming TKI resistance. It is worthy to note that PI3K and SIAH2 are potentially regulated by a common group of microRNAs in miR-30 family. Our bioinformatics analyses showed upregulated SIAH2 expression in NSCLC based on mass spectrometry data, explored its indirect interaction with PI3K and predicted their targeting microRNAs in common. We have also explored the potential role of miR-30 family in the modulation of PI3K-SIAH2 interaction in NSCLC.

## Introduction

Every year, about 1.8 million new lung cancer cases are diagnosed, claiming 1.6 million deaths globally. Non-small cell lung cancer (NSCLC) is a major form of lung cancer, comprising approximately 84% of all lung cancers. Although many treatment options, including targeted therapy, are available, the survival rate is still very low because of ineffective treatment outcome and acquired drug resistance. In Hong Kong, the survival rate of NSCLC is about 15% only (Ho, 2011). Although cigarette smoking has predominant association with lung cancer, non-smokers constitute a substantial minority of lung cancer patients. In the USA, 17,000–26,000 annual deaths of non-smokers from lung cancer account for the seventh leading cause of cancer mortality (Rivera and Wakelee, 2016). With the exposure to passive smoke and cooking fumes, non-smoking women represent an under-studied subset of NSCLC patients (Shen et al., 1998).

The current therapeutic approaches mainly target the molecular mechanisms of tumor cell growth caused by the aberrant activity of epidermal growth factor receptor (EGFR) (Camp et al., 2005). EGFR tyrosine kinase inhibitors (TKIs) are small molecules designed to bind to the tyrosine kinase domain of EGFR so that its phosphorylation and the subsequent receptor activation and signal transduction can be inhibited. However, the cancer progression remains because of the acquired resistance to EGFR TKIs (Yu et al., 2013). To date, no effective therapy is available for NSCLC patients with such resistance. Multiple molecular mechanisms of resistance have been explored, such as the activation of alternative receptor tyrosine kinases (RTKs) that bypass the EGFR signaling pathway, and the secondary mutations in EGFR T790M (Camp et al., 2005; Zhang et al., 2012). The resistance to anti-EGFR therapy may be caused by the activation of other RTKs, such as hepatocyte growth factor receptor (c-MET), Ron (a protein tyrosine kinase related to c-MET), platelet-derived growth factor receptor (PDGFR), and insulin-like growth factor receptor-1 (IGF-1R), which share the same downstream signaling cascade with EGFR pathway (Camp et al., 2005). The other activated RTKs are able to substitute the function of EGFR by activating overlapping signal transduction. The downstream molecule phosphoinositide-3-kinase (PI3K) shared by both EGFR and IGF-1R is associated with the EGFR TKIs resistance (Huang and Fu, 2015).

### PI3K Signaling Cascade Coupled with RTKs

Upon ligand binding, RTKs dimerize and phosphorylate each other. The adaptor proteins recruit the p85 regulatory subunit of Class IA PI3K heterodimer, which is expressed by PIK3R1, PIK3R2, PIK3R3, p85β, and p55γ, to the phosphorylated cytosolic RTK domains. The recruitment of p85 subunit results in a conformational change in the PI3K heterodimer that activates its p110 catalytic subunit, phosphorylates phosphatidylinositol 4,5-bisphosphate (PIP2) and converts PIP2 to phosphatidylinositol (3,4,5)-trisphosphate (PIP3). Protein kinase B (AKT) is then recruited by PIP3 to the cell membrane where the conserved serine residue of AKT is phosphorylated by TORC2. The phosphorylated AKT interact with a number of proteins participating in cell survival and metabolism (Manning et al., 2002).

Casitas B-lineage lymphoma (CBL) and PI3K are both downstream proteins of EGFR. CBL is an E3 ubiquitin ligase consisting of tyrosine kinase binding (TKB), RING finger domain and ubiquitin-associated domains. PI3K-phosphorylated protein activates TKB domain at the N-terminal of CBL and stimulates its ubiquitination activity. With response to this signal, RING finger domain recruits an E2 ubiquitin-conjugating enzyme, such as UBE2D2, and ubiquitin-associated domain transfers ubiquitin from the enzyme to targeted substrates for degradation. Such process may promote the ubiquitination of RTK by degradation and attenuates RTK signal transduction.

### SIAH2 in EGFR Pathway

The seven in absentia homolog 2 (SIAH2) is another E3 ubiquitin ligase that also mediates ubiquitination and subsequent proteasomal degradation of target proteins. In addition to AKT and PI3K, p38 mitogen-activated protein kinase (MAPK) are the downstream proteins of EGFR pathway. Major phosphorylation sites of SIAH2, S29, and T24, contains the SQ/TQ amino acid sequence motif, which is conserved for p38 MAPK phosphorylation. Hypoxia is a condition that promotes the activities of both SIAH2 and p38 MAPK (Nakayama et al., 2009). Under hypoxic condition, p38 MAPK actively phosphorylates and stabilizes SIAH2 in cytoplasm (Emerling et al., 2005; Khurana et al., 2006). However, the role of SIAH2 in NSCLC remains unclear.

### Post-transcriptional Regulation by MicroRNAs

MicroRNAs (miRNAs) represent a class of endogenous and non-coding RNAs whose mature forms are short fragments of 21–25 nucleotides long. To post-transcriptionally regulate the expression of their target genes, miRNAs bind to the miRNA response elements (MREs) of their messenger RNAs (mRNAs). MREs are the sequence motifs in 3′-untranslated region (3′-UTR) of mRNAs complementary to the targeting miRNAs. MiRNAs are not only able to regulate the expression of the target oncogenes or tumor suppressor genes in human tumors, but also to alter the acquired drug resistance and tumor progression so that the treatment response to targeted therapy is improved particularly in NSCLC (Medina and Slack, 2008; Weiss et al., 2008; Han et al., 2015; Yu and Cho, 2015; Sin et al., 2016). It was reported that the gefitinib-induced apoptosis and epithelial-mesenchymal transition of NSCLC cells were affected by miR-30b and miR-30c through the inhibition of some oncogenes, such as sarcoma viral oncogene homolog (SRC) (Skog et al., 2008). As a miRNA able to target multiple genes, it is interesting to examine whether miR-30 family plays a theranostic role in regulating PI3K and SIAH2 concurrently and mediating the potential PI3K–SIAH2 interaction in NSCLC.

## Materials and Methods

### Proteomic Analysis of Blood Samples

The plasma samples of this study were archived specimens. Among 105 patients randomly selected from pretreatment patients of Queen Elizabeth Hospital in Hong Kong, plasma samples were collected from 60 non-cancer patients and 45 adenocarcinoma patients. The non-cancer group includes 30 lung disease patients without known neoplastic tumor and 30 healthy volunteers free of any known acute or chronic illness. The adenocarcinoma group consists of 45 female nonsmokers (Eskandar et al., 2015). The plasma samples was fractionated and then profiled with the surface-enhanced laser desorption/ionization time-of-flight mass spectrometer (SELDI-TOF-MS), we analyzed the array on a Proteinchip PCS4000 Reader (Ciphergen Biosystems) with acquisition up to 200 kDa and generated the *m*/*z* spectra by averaging a total of 338 laser shots at an intensity of 195. The proteomic profile data from fractionation with pH 9 with cationic CM10 chip was used in this study.

### Peptide Prediction and Differential Expression Analysis

The molecular mass of SIAH2 is 34.615 kDa. It was predicted by Mascot MS/MS Ions Search (Matrix Science^1^) that a peptide with mass of 34461.667 Da could constitute 2–324 residues of SIAH2 and correspond to the *m*/*z* ratio 17230.8335 Da in the generated mass spectra. At this *m*/*z* ratio, we compared the intensity values of the adenocarcinoma group with that of the non-cancer group using independent sample *t*-test or its non-parameter counterpart depending on the normality of data. Differential expression of SIAH2 was indicated by *p* < 0.05 in the statistical test.

### Protein–Protein Interaction Search

We used Search Tool for Recurring Instances of Neighbouring Genes (STRING) to identify the potential interactions among PI3K, CBL, UBE2D2, and SIAH2. STRING hosts the information and corresponding evidence support of known and predicted protein–protein interactions in database and serves query functions and result visualizations. The interactions covered by STRING are not only experimentally-proved direct physical associations but also phylogenetic, genomic, or functional indirect associations. Evidence of identified interactions is supported by other primary databases, such as General Repository for Interaction Datasets (GRID). As of September 2016, STRING database includes the interaction information about 9,643,763 proteins from 2,031 organisms (Franceschini et al., 2013).

### MiRNA Target Prediction

TargetScan was used to predict the miRNAs that concurrently regulate PI3K, CBL, UBE2D2, and SIAH2. In the prediction process, TargetScan searched for the miRNAs whose seed regions match the conserved 8-mer, 7-mer, and 6-mer sites of the target genes’ mRNAs (Lewis et al., 2005). We did not limit the broadly conserved, conserved, and poorly conserved but confidently annotated microRNA families and included no other microRNA annotations in our search.

## Results

For testing the normality of data, Shapiro–Wilk test was performed to SELDI-TOF-MS intensity data of both non-cancer and adenocarcinoma groups. Normality cannot be assumed in non-cancer group (*p* < 0.0001) and adenocarcinoma group (*p* = 0.001). So, the difference between two groups was examined using non-parametric test, independent samples Mann–Whitney *U* test. It was found that the intensities of *m*/*z* = 17230.8335 Da were significantly different between two groups (*p* = 0.002, median in non-cancer = 0.1898, median in adenocarcinoma = 0.3075, see **Supplementary Materials**). This implies that the protein expression of SIAH2 is significantly upregulated in adenocarcinoma.

**FIGURE 1:**
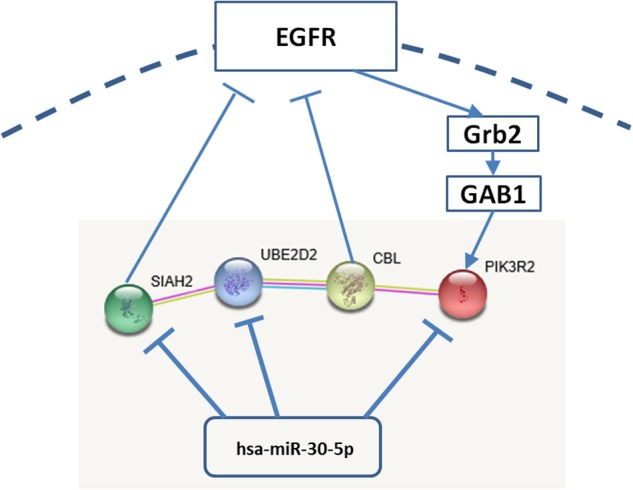
**Interactions among PI3K, CBL, UBE2D2, SIAH2, and miR-30-5p.** Grb2: growth factor receptor-binding protein 2; GAB1: GRB2-associated-binding protein 1.

Search Tool for Recurring Instances of Neighbouring Genes predicted the interactions among PI3K, CBL, UBE2D2, and SIAH2 (**Figure 1**). All the interactions were supported by experimental results and co-mentioning in literature. The CBL-UBE2D2 interaction was further supported by the pathways curated from third-party databases, such as Kyoto Encyclopedia of Genes and Genomes (KEGG). Datasets relevant to these interactions are shown in **Table 1**. Although there was no evidence showing that PIK3R2 directly interact with SIAH2, TargetScan predicted that miR-30-5p family concurrently targets them. The involved members of miR-30-5p family include miR-30a-5p, miR-30b-5p, miR-30c-5p, miR-30d-5p, and miR-30e-5p. The type of pairing site between PIK3R2 and miR-30-5p family members is 8mer and the target site is conserved among mammals. The type of pairing site between SIAH2 and miR-30-5p family members is 7mer-A1 and the target site is conserved among a number of vertebrates. It is interesting to note that miR-30-5p family also targets UBE2D2.

**Table 1:** Protein–protein interactions detected by assays and the corresponding hosting databases.

Protein 1	Protein 2	Database	Detected by
PIK3R2	CBL	Intact	Anti tag coimmunoprecipitation assay (psi-mi: “MI:0007”), tandem affinity purification assay (psi-mi: “MI:0676”), anti bait coimmunoprecipitation assay (psi-mi: “MI:0006”)
PIK3R2	CBL	Intact, pride	Cross-linking assay (psi-mi: “MI:0030”)
PIK3R2	CBL	GRID	Affinity capture-western assay, affinity capture-MS assay
CBL	UBE2D2	dip	Surface plasmon resonance and X-ray crystallography assays
CBL	UBE2D2	GRID	Reconstituted complex assay
UBE2D2	SIAH2	GRID	Reconstituted complex assay

## Discussion and Conclusion

This work investigated the roles of PIK3R2, CBL, UBE2D2, SIAH2, and miR-30-5p family that may be related to the resistance to anti-EGFR therapy. Among these factors, SIAH2 has not been studied sufficiently and its role in cancer treatment remains unclear. In the comparison between plasma samples of independent groups, we showed the significant upregulation of SIAH2 protein in NSCLC. Moreno et al. (2015) obtained similar result but the SIAH2 expression in lung cancer was compared with that in adjacent normal tissues. Sun et al. (2016) found that SIAH2 was overexpressed in human breast cancer tissues. Our finding suggests that SIAH2 may be a novel theranostic target in the circulation of NSCLC patients.

Protein–protein interaction database search revealed the experimental proofs using the reconstituted complex assays that the E2 ubiquitin-conjugating enzyme, UBE2D2, interacts with both CBL and SIAH2, E3 ubiquitin ligases. CBL ubiquitylates active RTKs for degradation and attenuates directly the receptor signaling, including that mediated by EGFR (Joazeiro et al., 1999). The potential theranostic role of CBL has been shown in Klapper et al. (2000) that some antibodies may enhance directing HER-2 to CBL-regulated proteolysis. Similar to CBL, SIAH2 ubiquitylates protein for degradation. However, there is no evidence at the moment showing that the ubiquitination of EGFR is related to SIAH2. On the other hand, it was shown in Szargel et al. (2009) that SIAH2 is responsible for the ubiquitination of alpha-synuclein and synphilin-1 in Lewy bodies of PD patients. The only association with EGFR pathway is that both SIAH2 and MAPK are sensitive to hypoxia and MAPK, a downstream protein of EGFR can activate SIAH2 by phosphorylation (Nakayama et al., 2009).

TargetScan predicted that mRNAs of PIK3R2, UBE2D2, and SIAH2 are concurrently targeted by miRNA-30-5p family. These three RNA transcripts sharing the same conserved MREs are called competing endogenous RNA (ceRNA). CeRNAs interact indirectly by competing for their common targeting miRNAs in cells. The up-regulation of a ceRNA could indirectly promote the expression of the other ceRNAs (Marques et al., 2011). The molecular mechanism, through which the ceRNAs are co-regulated, paves the undiscovered cascades in the signaling pathway. Such co-regulation can be detected by the co-expression of ceRNAs. It was shown that PTEN and its putative ceRNAs are co-expressed in prostate cancer and glioblastoma (Tay et al., 2011). The ceRNA crosstalk is susceptible to the miRNA:target concentration ratio and, in other words, mediated by the targeting miRNAs (Bosson et al., 2014).

Among the miRNA-30-5p family members, microRNA-30a expression was found negatively associated with the malignant potential of various NSCLC cell lines, positively with the expression of E-cadherin, which is an epithelial marker, and negatively with the expression of N-cadherin, which is a mesenchymal marker. In other words, the epithelial-to-mesenchymal transition could be inhibited by miR-30a (Kumarswamy et al., 2012). Based on the above evidence, interaction of PIK3R2-SIAH2 ceRNAs and its mediator, miRNA-30-5p family, can be considered as a potential treatment target and theranostic panel in NSCLC, particularly adenocarcinoma. The treatment effect can be examined by intravenous injection of Lipofectamine reagents of miRNA-30-5p inhibitor/mimic in xenograft model and clinical trials.

## Ethics Statement

The study was exempt from ethics approval because only the archived plasma samples were analyzed. No prospective study was performed.

## Author Contributions

LC wrote this paper, provided the research idea and performed the analysis of this work. FW advised about the association of microRNA-30 family with non-small cell lung cancer and the bioinformatics analysis. LW advised about the bioinformatics analysis. FM advised about the functional role of microRNA-30 family in non-small cell lung cancer. SW advised about the molecular and genomics perspective for theranostic markers in non-small cell lung cancer. JA advised about the clinical perspective for theranostic markers in non-small cell lung cancer. SY advised about the cellular self-homeostasis under stress, particularly hypoxic response. WC provided the proteomics data and advised about the clinical diagnostic need and the bioinformatics analysis.

## Conflict of Interest Statement

The authors declare that the research was conducted in the absence of any commercial or financial relationships that could be construed as a potential conflict of interest.

## Footnotes

1 http://www.matrixscience.com/

## Supplementary Material

TABLE S1 **Intensities of m/z = 17230.8335 Da across 105 blood samples**.
